# Use of CRISPR‐Cas tools to engineer *Trichoderma* species

**DOI:** 10.1111/1751-7915.14126

**Published:** 2022-07-31

**Authors:** Ying Wang, Hongyu Chen, Liang Ma, Ming Gong, Yingying Wu, Dapeng Bao, Gen Zou

**Affiliations:** ^1^ Shanghai Key Laboratory of Agricultural Genetics and Breeding, Institute of Edible Fungi Shanghai Academy of Agricultural Sciences Shanghai China; ^2^ Key Laboratory for Quality Improvement of Agricultural Products of Zhejiang Province, College of Advanced Agricultural Sciences Zhejiang A&F University Lin'an Hangzhou China

## Abstract

Given their lignocellulose degradability and biocontrol activities, fungi of the ubiquitously distributed genus *Trichoderma* have multiple industrial and agricultural applications. Genetic manipulation plays a valuable role in tailoring novel engineered strains with enhanced target traits. Nevertheless, as applied to fungi, the classic tools of genetic manipulation tend to be time‐consuming and tedious. However, the recent development of the CRISPR‐Cas system for gene editing has enabled researchers to achieve genome‐wide gene disruptions, gene replacements, and precise editing, and this technology has emerged as a primary focus for novel developments in engineered strains of *Trichoderma*. Here, we provide a brief overview of the traditional approaches to genetic manipulation, the different strategies employed in establishing CRSIPR‐Cas systems, the utilization of these systems to develop engineered strains of *Trichoderma* for desired applications, and the future trends in biotechnology.

## Graphical abstract

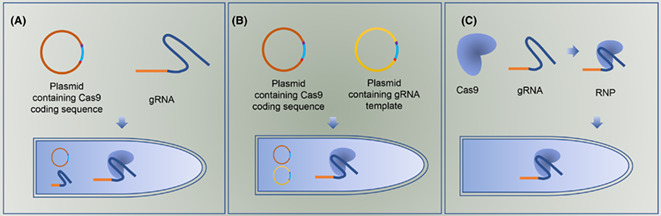

## INTRODUCTION

The genus *Trichoderma* comprises a range of rhizocompetent soil‐dwelling moulds with parasitic or saprotrophic lifestyles (Kubicek et al., 2011). They can exploit a diverse range of substrates and thrive on the resources provided by plants, other fungi, and animals. In addition, it has recently been established that members of this genus are widely distributed not only in terrestrial ecosystems but also in marine habitats (Li, Lin et al., 2021; Li, Liu et al., 2021). Given their broad host range and adaptability, species of *Trichoderma* have found widespread industrial and agricultural applications as a source of enzyme preparations (Zou, Bao et al., 2021; Zou, Li et al., 2021; Zou, Xiao et al., 2021) and biological control agents (Tomico‐Cuenca et al., 2021). Moreover, they are important renewable natural resources with high economic value and application potential because of their ability to grow and reproduce on inexpensive substrates, including agricultural and forestry wastes (Zain Ul Arifeen et al., 2019).

Designated as a generally recognized as safe (GRAS) organism by the U.S. Food and Drug Administration, *T. reesei* has been used as a model strain in the cellulase preparation industry for over half a century (Bischof et al., 2016). Some *Trichoderma* fungi, including *T. harzianum*, *T. virens*, and *T. longibrachiatum*, have also been used for the production of extracellular enzymes (Dong et al., 2022; Papzan et al., 2021; Zeng et al., 2016). Intriguingly, a commercial enzyme preparation called Lysing Enzymes from *T. harzianum* (Sigma®, L1412) has been commonly used for the preparation of fungal protoplasts (Zeng et al., 2016). *Trichoderma* spp., has been exploited as a biological plant protection agent for nearly a century, which is another important application. The most effective biocontrol properties were mainly attributed to *T. virens* (de Souza Maia Filho et al., 2017), *T. harzianum* (Zhang et al., 2016), *T. koningii* (Gajera et al., 2016), *T. pseudokoningii* (Zavala‐Gonzalez et al., 2016), *T. longibrachiatum* (Sridharan et al., 2021), *T. asperellum* (Xian et al., 2020), and *T. viride* (Kumar et al., 2021).

In recent years, a range of *Trichoderma* applications has undergone considerable expansion, facilitated by rapid ongoing developments in the field of biotechnology (Benitez et al., 2004; Bischof et al., 2016; TariqJaveed et al., 2021; Xie et al., 2021; Zhang et al., 2021). Notable in this regard has been the advances made in genetic modification and the generation of massive amounts of genomic data for this genus, including *T. reesei* (Martinez et al., 2008), *T. atroviride* (Kubicek et al., 2011), *T. virens* (Kubicek et al., 2011), *T. longibrachiatum* (Kubicek et al., 2011), *T. harzianum* (Steindorff et al., 2014), and *T. asperellum* (Druzhinina et al., 2018; Kubicek et al., 2019; Li, Lin et al., 2021; Li, Liu et al., 2021). Industrial, agricultural, and even pharmaceutical biotechnologies are particularly reliant on these tools to meet the increasing demands and augment the number and diversity of *Trichoderma*‐derived proteins, metabolites, biomolecules, and chemical products (Mukherjee et al., 2013). These advances are of particular relevance in the case of multicellular organisms such as *Trichoderma*, given that genetic modification based on traditional methods is rarely as straightforward as that in unicellular microorganisms (including bacteria and yeasts), owing to complex cellular differentiation, thick chitinous cell walls, and lack of self‐replicating vectors (Jiang et al., 2013).

Among the more recent advances in genetic engineering, the use of artificially engineered nucleases is an effective approach for investigating the function of genes and proteins (Tomico‐Cuenca et al., 2021). In *Trichoderma*, these nuclease‐based editing tools mainly include clustered regularly interspaced short palindromic repeats (CRISPR) RNA‐guided Cas9 (CRISPR‐Cas9; de Souza Maia Filho et al., 2017) and transcription activator–like effector nucleases (TALENS; Liu et al., 2015). Compared to TALENs and other editing strategies, CRISPR‐Cas gene editing methods are currently the most efficient, convenient, and widely used for genome engineering (Burgess, 2013).

The discovery and manipulation of CRISPR and Cas genes has accelerated recent developments in flexible, cost‐effective genomic engineering toolkits based on the programmable targeting of CRISPR‐Cas technologies (Deveau et al., 2010). Researchers have employed CRISPR‐Cas to modify the genomes of a diverse range of organisms by introducing double‐strand breaks (DSBs; Burgess, 2013), which activate sequence variations (insertions, deletions, and rare substitutions near DNA cleavage sites) conducted by non‐homologous end joining (NHEJ); precise sequence alterations conducted by homology‐directed repair (HDR) with the endogenous repair pathways (artificial supply of repair template; Horwitz et al., 2015). Using CRISPR‐Cas tools, it is possible to simultaneously edit multiple loci, highlighting the potential utility of this technique as an extensible system for versatile genome‐wide engineering (Cong et al., 2013; Li et al., 2013; Liu & Fan, 2014). Most applications of the CRISPR‐Cas system also have direct utility and relevance with respect to *Trichoderm*a, for which CRISPR‐Cas can be applied to augment and/or enhance pre‐existing genetic engineering platforms (Liu et al., 2015). To date, researchers have constructed CRISPR‐Cas tools based on diversification strategies and have applied them to functional gene identification, strain modification, and other fields in *T. reesei* (Bodie et al., 2021; Chai et al., 2022; de Souza Maia Filho et al., 2017; Hao & Su, 2019; Li, Lin et al., 2021; Li, Liu et al., 2021; Liu et al., 2015; Rantasalo et al., 2019; Wu, Chen, Huang, et al., 2020; Wu, Chen, Qiu, et al., 2020; Zou, Bao et al., 2021; Zou, Li et al., 2021; Zou, Xiao et al., 2021). Nevertheless, some *Trichoderma* species, including *T. harzianum* (Vieira et al., 2021), *T. atroviride* (Primerano, 2021), and the unidentified species *Trichoderma* sp. LF328 (Vidgren et al., 2020), have only built CRISPR systems, and the rest have not yet been reported to attempt genome editing. For these *Trichoderma* species, the leading research progress in *T. reesei* can serve as a reference paradigm.

In this short review, we focus on the most recent evolution and applications of CRISPR‐Cas‐mediated genomic engineering for gene editing and its imminent implications regarding the industrial application of *Trichoderma*, mainly including (i) transformation methods, (ii) Cas nuclease and sgRNA delivery strategies, and (iii) applications of CRISPR‐Cas genome editing in *Trichoderma* species.

## TRANSFORMATION METHODS

At present, neither the initial proofs of concept nor the practical applications of CRISPR‐Cas in fungi, including *Trichoderma*, are completely independent of the traditional tools of genetic manipulation, which are responsible for introducing two essential components of the CRISPR‐Cas system: Cas genes/proteins and guide RNAs (gRNAs) (or templates). Consequently, classical genetic transformation technology remains an essential requirement for the development of CRISPR‐Cas tools.

**FIGURE 1:**
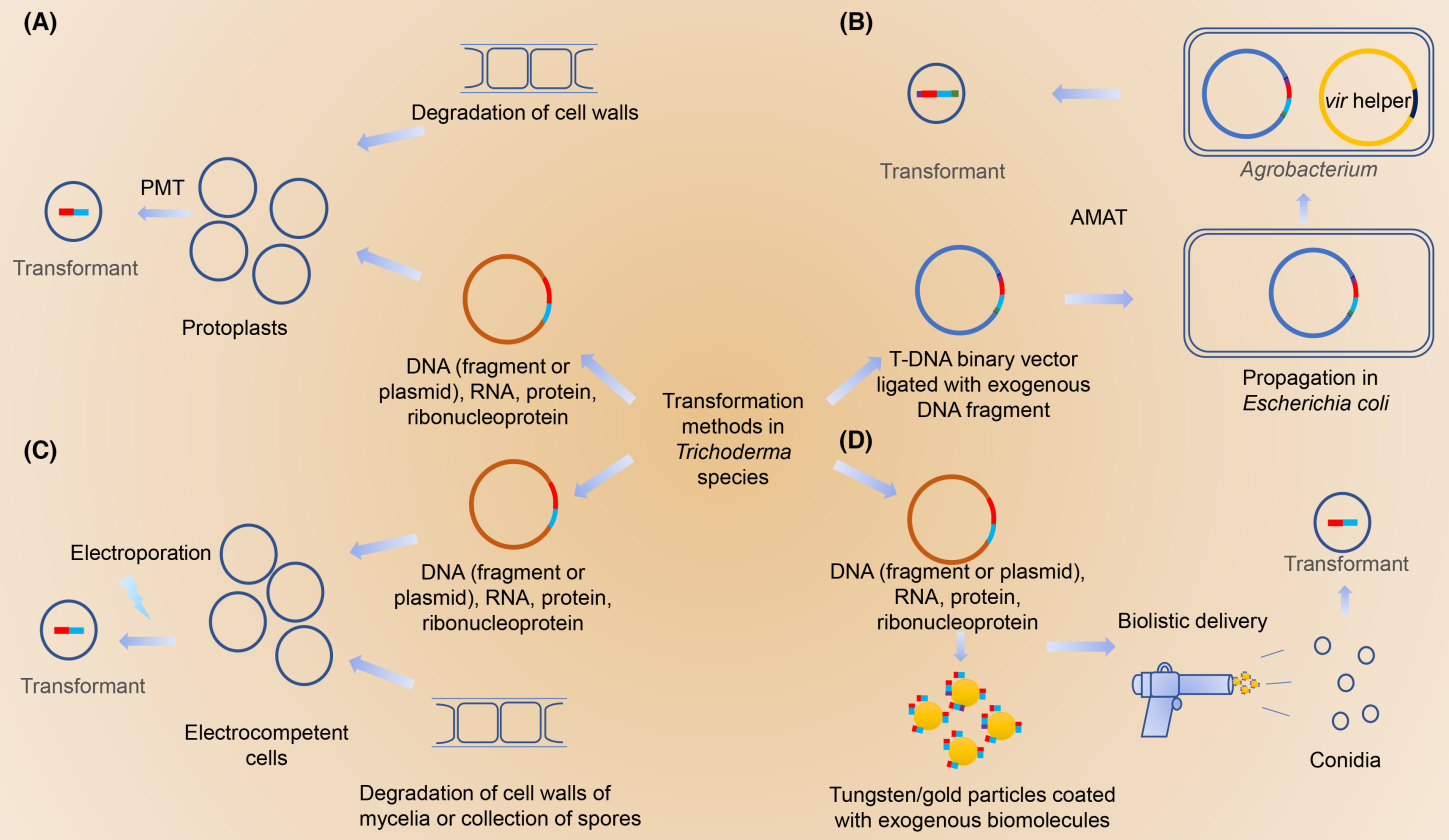
Schematic diagrams of classical transformation strategies in *Trichoderma* species. (A) Polyethylene glycol (PEG)–mediated transformation of protoplasts (PMT). (B) *Agrobacterium tumefaciens*‐mediated transformation (AMAT). (C) Electroporation. (D) Biolistic delivery. In general, PMT (A), electroporation (C), and biolistic delivery (D) are theoretically compatible with various biomolecules such as linear or circular DNA, RNA, protein, ribonucleoprotein. However, plasmid, ligated with a selectable marker (red) and an expression cassette of a gene of interest (light blue), is the most common biomolecule for transformation. In AMAT (B), the biomolecule is be limited to DNA fragments which is flanked by left border (purple) and right border (green). These plasmids all contain kanamycin, ampicillin, or other antibiotic resistance in their skeletons, enabling screening and propagation in *Escherichia coli*.

A diverse range of genetic manipulation methods have been applied in engineering *Trichoderma* (Figure 1; Li et al., 2017), the most common of which is the polyethylene glycol (PEG)–mediated transformation of protoplasts (PMT; Figure 1A; Penttilaa et al., 1987), *Agrobacterium tumefaciens*‐mediated transformation (ATMT; Figure 1B; de Groot et al., 1998), electroporation (Figure 1C; Sanchez‐Torres et al., 1994), and biolistic delivery (Figure 1D; Lorito et al., 1993). The transformation rates achieved using these approaches tend to differ depending on the technique applied and the target *Trichoderma* species.

### PMT

Transformation of fungi tends to be hampered to varying extents by their multicellular structure and thick cell walls; to overcome these barriers, cocktails of fungal cell wall‐degrading enzymes are used to generate protoplasts via the enzymolysis of hyphal cell walls (Figure 1A). Being deprived of its protective cell wall, protoplasts are preserved in a hyperosmotic solution, generally high concentrations of either sucrose (0.6 M) or sorbitol (1.2 M) with a slightly basic pH value (pH 7.5), to prevent swelling and rupture. During transformation, protoplasts, as receptor cells, are transformed with DNA (or other biomolecules, including circular/linearized plasmids, RNAs, proteins, and ribonucleoproteins) using 25% (w:v) PEG 6000 and 50 mM calcium chloride. Given its simplicity, relative rapidity, and high yields, this method has become the technique of choice for the transformation of a number of *Trichoderma* species (Cai et al., 2021; Cardoza et al., 2006; Herrera‐Estrella et al., 1990). After optimizing the process, the transformation efficiency reached 200 ~ 800 colonies per microgram of DNA in *T. reesei* (Herrera‐Estrella et al., 1990).

### AMAT

AMAT, as a well‐established simple and versatile method, is based on the natural infectivity of *A. tumefaciens* towards plants and the transformation of host genomes using a partial Ti vector (Zeilinger, 2004). This process can be readily harnessed for the genetic manipulation of plants and have been adapted to introduce exogenous genetic material in filamentous fungi. Generally, a modified binary vector system is required for the transport of foreign genes into diverse recipient cells (conidia, protoplasts, and even mycelia; Figure 1B). Using this binary system, the T‐DNA and *vir* regions are inserted into two independent plasmids. A selectable marker gene and an exogenous gene of interest are introduced between the T‐DNA borders, which are essential for the transformation and release of the DNA fragments therein (Michielse et al., 2004). The released fragments are then randomly inserted (DNA fragments without HDR) or homologously recombined (DNA fragments with HDR) into the genome. The use of this method has been reported with respect to gene disruption in *T. atroviride* (Zeilinger, 2004) and was subsequently successfully developed for the transformation of *T. reesei* using *hph* (encoding hygromycin B phosphotransferase) as the selectable marker gene (Zhong et al., 2007). Furthermore, in 2019, a modified ATMT method using two different *A. tumefaciens* strains was reported that could be used to simultaneously introduce two plasmids in a single step (Wu, Chen, Huang, et al., 2020; Wu, Chen, Qiu, et al., 2020). Although AMAT is another common transformation method, it resulted in a lower efficiency of DNA integration and less stable transformants when the ATMT and PMT methods were compared in four different *Trichoderma* species (Cardoza et al., 2006). The inefficiency of AMAT may be due to the fact that the two methods used different receptor cells. In *T. reesei*, AMAT efficiency increased 10‐ to 50‐fold in protoplasts compared to conidia (Zhong et al., 2007).

### Electroporation

In this technique, electric current pulses are used to puncture micropores in the cell membrane, thereby enabling foreign DNA to penetrate the membrane and enter the cell. It is essential to select an appropriate field intensity to restore the cell viability. Excessively powerful electric fields can be lethal, owing to irreversible damage to the cell membrane (Li et al., 2017). Electroporation‐based genetic manipulation has been established for *T. harzianum* (Goldman et al., 1990). The process of producing competent cells suitable for electroporation was similar to that used to generate protoplasts. Competent cells were primed for DNA penetration using an electric pulse with or without PEG 6000 (optional; Figure 1C; Goldman et al., 1990, Cai et al., 2021). Conidial spores have also been used for receptor cell electroporation (Kim & Miasnikov, 2013). Perhaps, it is the most promising protocol for delivering DNA to *Trichoderma* fungi because of its ease, efficiency, and reduced hands‐on time. However, there is a lack of clarity regarding the variables of spore electroporation and which conditions are likely to achieve the highest transformation efficiency (Kim & Miasnikov, 2013).

### Biolistic bombardment

In biolistic bombardment of cells, DNA‐coated gold or tungsten particles are fired into the cells at a high speed (Figure 1D). It is a rapid and convenient method that does not require the preparation of osmotic pressure‐sensitive protoplasts or time‐consuming co‐culture with *A. tumefaciens*; however, it requires the initial purchase of additional expensive equipment and reagents (Li et al., 2017). To date, biolistic bombardment has been adapted for the transformation of a number of *Trichoderma* species, including *T. harzianum* (Lorito et al., 1993), *T. longibrachiatum*, and *T. reesei* (Hazell et al., 2000), the efficiency of which depends primarily on the following three parameters: scattering distance of particles prior to impacting the cells, vacuum intensity in the cavity, and density and size of particles. Biolistic transformations in fungi are reportedly less efficient than protoplast uptake (Hazell et al., 2000). The transformation efficiency reached only 35–40 colonies per microgram of DNA (linear or circular plasmid DNA) in *T. reesei* (Te'o et al., 2002).

In summary, in *Trichoderma*, PMT is simple, efficient, does not require complicated equipment, and is suitable for a variety of biomolecules. This has become the most common transformation strategy for delivering the CRISPR system (Table 1).

**TABLE 1:** CRISPR‐Cas systems established in *Trichoderma* species

Species	Strategies			Editing type and application	Efficiency	Reference
	Cas	gRNA	Transformation method			
*T. reesei*	*T. reesei* codon optimized	Transcribed in vitro	AMAT (Cas9) and PEG (gRNA)	Single/multiple gene disruption or replacement;	4.2% (triplex) –100% (single)	Liu et al. (2015)
*T. reesei*	*T. reesei* codon optimized	Transcribed in vitro	PMT	Single‐gene replacement; gene function investigation	N/A	Liu, Chen et al. (2017); Liu, Wang et al. (2017)
*T. reesei*	RNP	RNP	PMT	Single‐gene disruption	3.5% (*cel3c*) –14.8% (*cbh1*)	Hao and Su (2019)
*T. reesei*	RNP	RNP	PMT	Single/triple gene replacement; chassis modification; strain engineering	6% (double) –23% (single); 12% (triple)	Rantasalo et al. (2019)
*T. reesei*	*T. reesei* codon optimized	U6 snRNA promoter	AMAT	Single‐gene disruption	1–10%	Wu, Chen, Huang, et al. (2020); Wu, Chen, Qiu, et al. (2020)
*Trichoderma* sp.	RNP	RNP	PMT	Single‐gene replacement; strain engineering	100%	Vidgren et al. (2020)
*T. reesei*	RNP	RNP	PMT	Single/multiple gene disruption or replacement; marker‐free gene disruption	7.4% (marker free) –100% (single); 10.0% (triple)	Zou, Bao et al. (2021); Zou, Li et al. (2021); Zou, Xiao et al. (2021)
*T. reesei*	*Aspergillus niger* codon optimized	5S rRNA promoter	PMT	Single‐gene disruption	6.7% (heterologous 5S rRNA promoter) –36.7% (native 5S rRNA promoter)	Wang et al. (2021)
*T. reesei*	RNP	RNP	PMT	Single‐gene replacement; marker‐recycled iterative replacement; chassis modification	N/A	Chai et al. (2022)
*T. reesei*	*Aspergillus niger* codon optimized	5S rRNA promoter	PMT	Single‐gene disruption	6.7% (heterologous 5S rRNA promoter) –36.7% (native 5S rRNA promoter)	Wang et al. (2021)
*T. reesei*	*T. reesei* codon optimized	U6 snRNA promoter with 1st intron	PMT	Single‐gene replacement; gene function investigation	N/A	Bodie et al. (2021)
*T. harzianum*	*T. harzianum* codon optimized	*T. reesei* derived Pol II *tef1* promoter	Biolistic transformation	Single‐gene disruption	N/A	Vieira et al. (2021)
*T. atroviride*	RNP	RNP	PMT	Single/double gene disruption	N/A	Primerano (2021)

## Cas nuclease and gRNA delivery strategies

**FIGURE 2:**
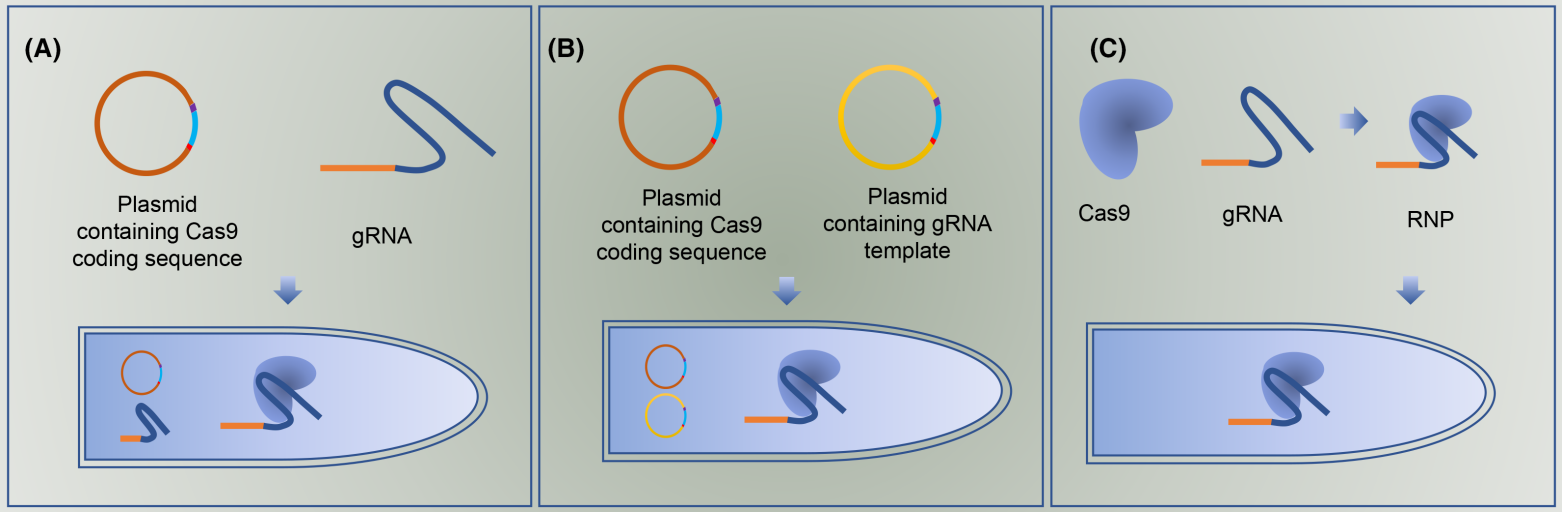
Schematic diagrams of CRISPR‐Cas systems in *Trichoderma* species. (A) Cas9‐expressing chassis with gRNA in vitro. Cas9‐expression cassette containing the codon‐optimized *cas9* gene with NLS (light blue) is controlled by an appropriate promoter (red) and terminator (purple). gRNA (yellow: crRNA spacer sequence; sapphire: tracrRNA) is transcribed using T7 promoter in vitro and then is transformed with Cas9‐expressing chassis cells. (B) Plasmid‐based CRISPR‐Cas. Cas9‐expression cassette and gRNA‐transcription cassette can be constructed in a plasmid or separately in two plasmids. (C) RNP‐based CRISPR‐Cas. RNP is pre‐assembled with Cas9 and gRNA in vitro and transformed within cells.

The different transformation methods developed for *Trichoderma* can be modified to transform cells with different biomolecules, and a diverse range of strategies have been adopted to introduce Cas nucleases and guide RNAs (gRNAs) into *Trichoderma* (Table 1). Here, we summarize three common strategies employed for the delivery of Cas nuclease and gRNA: Cas9 in vivo and gRNA in vitro (Cas9‐expressing chassis with gRNA in vitro; Figure 2A), both Cas and gRNA in vivo (plasmid‐based CRISPR‐Cas; Figure 2B), and both Cas9 and gRNA in vitro [ribonucleoprotein (RNP)‐based CRISPR‐Cas] (Figure 2C).

These three strategies will be discussed in detail below.

### Cas‐expressing chassis with gRNA in vitro

*Trichoderma* genes show a high GC bias at the codon wobble position (http://www.kazusa.or.jp/codon/). Unlike *Saccharomyces cerevisiae*, the codon optimized *cas9* gene for human cells does not function in many *Trichoderma* fungi (Liu et al., 2015; Tomico‐Cuenca et al., 2021; Zou & Zhou, 2021). Thus, the initial strategy used to establish the CRISPR‐Cas system in *T. reesei* involved the expression of codon‐optimized Cas9 nuclease (*Streptococcus pyogenes*) in vivo (Liu et al., 2015, Zou & Zhou, 2021; Table 1). Transcription of gRNA (10–50 μg of gRNA for 10^6^ protoplasts) using the T7 promoter in vitro (Figure 2A) was also the earliest established CRISPR‐Cas genome editing platform used for filamentous fungi and has been extensively applied in the engineering of a range of fungi (Chen et al., 2017, Zheng et al., 2017), including the basidiomycete fungus *Ganoderma lucidum* in which the in vitro–transcribed gRNA is introduced into protoplasts expressing the Cas nuclease (cellular host used as a recipient for further engineering) via PEG‐mediated transformation (Table 1; Qin et al., 2017). Depending on the fungal genomic sequence information, genome engineering, including multiplexing mutations and knockin/knockout, can be implemented with high efficiency [4.2% (triple loci) ~100% (single locus)] by modifying a 20‐bp protospacer of gRNAs corresponding to a target gene sequence (Liu et al., 2015). Consequently, this optimized CRISPR‐Cas strategy can save time and labor without the need to identify suitable RNA promoters for gRNA transcripts or repeatedly transform the Cas‐expressing plasmid. Moreover, compared with the continuous in vivo transcription of gRNA, transiently transforming gRNAs reduces the risk of off‐target modification (Liu et al., 2015).

### Plasmid‐based CRISPR‐Cas

This is a conventional strategy that requires the construction of plasmids for Cas expression and gRNA transcription (Figure 2B). In this approach, Cas expression boxes (codon‐optimized *cas* gene with nuclear localization signal sequence [NLS] controlled by an appropriate promoter and terminator) are either integrated into the genome or self‐replicating plasmids (Schuster & Kahmann, 2019). RNA polymerase III promoters, such as SNR52 and U6 snRNA promoters, have been used to transcribe gRNAs in fungi (DiCarlo et al., 2013; Liu et al., 2019). Although both of these promoters are conserved in eukaryotes, prediction of promoters is difficult using bioinformatics, owing to the diversity of canonical splice sites and branch site motifs (Canzler et al., 2016). Both the 5S rRNA (Wang et al., 2021) and U6 snRNA promoters (Bodie et al., 2021; Wu, Chen, Huang, et al., 2020; Wu, Chen, Qiu, et al., 2020) were able to transcribe gRNA in *T. reesei*. Although RNA polymerase III was initially believed to mediate microRNA transcription, circumstantial evidence suggests that the RNA polymerase II promoter is also responsible for microRNA transcription (Lee et al., 2004). In *T. harzianum*, the promoter *tef1* derived from *T. reesei* has been used to control gRNA transcription(Vieira et al., 2021).

### RNP‐based CRISPR‐Cas

Continuous Cas9 expression in vivo has been reported to cause unfavourable phenotypes such as reduced growth (Enkler et al., 2016) and even lethal effects in some organisms (Jiang et al., 2017). Besides, unexpected off‐target events often result due to two major factors when there is long‐term presence of Cas and gRNA within cells: less stringent recognition of protospacer adjacent motif flanking the target sequence and tolerance to target DNA‐gRNA mismatch (Kang et al., 2022). Therefore, researchers have recently developed a strategy designed around an in vitro pre‐assembled Cas‐gRNA complex for transient genome editing (Figure 2C; Kim et al., 2014). Using this approach in *T. reesei* QM9414, a pre‐assembled RNP of Cas9 (recombinant Cas9 expressed in *Escherichia coli*) and gRNA (transcribed by the T7 promoter in vitro), co‐transformed with the *pyr4* gene (syn. *ura3*, encoding orotidine‐5′‐phosphate decarboxylase) as a selective marker using PMT, has been used to disrupt the major cellulase gene *cbh1* (14.8%) and *cel3c* (3.5%; Hao & Su, 2019; Table 1). To further enhance editing efficiency, the additional use of the detergent Triton X‐100 has been reported to facilitate RNP penetration of the protoplast membrane in *T. reesei* and increase the editing efficiency to 100% for single‐gene disruption (Table 1). In the control group without Triton X‐100, only half of the correctly edited transformants were obtained (Zou, Bao et al., 2021; Zou, Li et al., 2021; Zou, Xiao et al., 2021). The enhanced CRISPR‐Cas9 ribonucleoprotein method has been adapted to a variety of fungi such as *Aspergillus oryzae*, *Cordyceps militaris*, and *Claviceps purpurea* (Yu et al., 2022; Zou, Bao et al., 2021; Zou, Li et al., 2021; Zou, Xiao et al., 2021). Multiplex editing requires the introduction of mixed RNPs within cell via, respectively, designing multiple gRNAs targeting different loci. However, it is usually low in efficiency (10.0% for triple genes) due to the limited receptivity of a fungal cell to exogenous biomolecules (Zou, Bao et al., 2021; Zou, Li et al., 2021; Zou, Xiao et al., 2021; Table 1). This suggests that more (≥4 loci) gene editing may require tactical improvements such as employing CRISPR‐Cas12a which does not require tracrRNA in crRNA processing and performs much easier in multiplex targeting (Paul & Montoya, 2020). The results obtained using this approach indicate that the direct introduction of an RNP complex into fungal cells is an optimal strategy for rapid, simple, and precise genomic engineering, with considerable potential for multiple applications in functional genomics. Moreover, it can be used to minimize off‐target events and cytotoxicity associated with the continuous expression of Cas nuclease in cells (Foster et al., 2018). This strategy also offers a promising gene‐engineering approach for completely exogenous DNA‐free solutions. Eradication of transgenic integration, DNA fragment insertion, and resistance marker selection in engineered mutants is highly accessible for public acceptance of genome edited organisms (Kanchiswamy, 2016). In conjunction with previously established molecular biology tools, this genome engineering technology represents a potentially powerful approach for the genetic manipulation of *Trichoderma* (Primerano, 2021; Rantasalo et al., 2019) and undoubtedly other fungi, thereby contributing to the progress in fungal studies on strain improvement and functional genomics (Zou, Bao et al., 2021; Zou, Li et al., 2021; Zou, Xiao et al., 2021).

In summary, the editing efficiency of different strategies generally depends on the total amount of Cas9, gRNA, or RNP in the cells (Table 1). Therefore, the promoter is critical for the expression of Cas9 and transcription of gRNA for the in vivo strategy (Table 1). In *T. reesei* C30‐cc (Cas9‐expressing chassis with inducible promoter pcbh1), gene editing was conditionally implemented by inducers (lactose or cellulose) or repressors (glucose; Liu et al., 2015). Similarly, the heterologous 5S rRNA promoter of *A. niger* showed only 6.7%, whereas the native promoter increased the editing efficiency to 36.7% in *T. reesei* (Wang et al., 2021). In addition to the dosage of RNP affecting editing efficiency, Triton X‐100 dramatically increased the number of edited mutants using the RNP transformation procedure, which could be attributed to the greatly improved efficiency (3.33‐fold) of RNP penetration by improving cell membrane permeability (Zou, Bao et al., 2021; Zou, Li et al., 2021; Zou, Xiao et al., 2021). The activity of two major pathways for the repair of Cas9‐induced DSBs is another important factor that affects editing efficiency (Rantasalo et al., 2019). The *T. reesei* strain containing the *mus53* deletion (increased the rate of HDR by suppressing NHEJ pathway) exhibited higher efficiency (12% for triple genes) in multiplexed editing (Rantasalo et al., 2019).

Although all the currently reported CRISPR‐Cas systems are based on Cas9 in *Trichoderma*, other Cas nucleases with diverse PAM motifs are probably compatible with *Trichoderma* species (Paul & Montoya, 2020). CRISPR nucleases with longer PAM are expected to cause fewer off‐target events. However, due to more stringent requirements of PAM sequences, it will reduce the number of practicable target sites (Kaya et al., 2016). The recent development of prime editing and base editor provides more potential strategies for engineering *Trichoderma* genome (Anzalone et al., 2019; Zhang et al., 2022).

## APPLICATIONS OF CRISPR‐CAS GENOME EDITING IN *Trichoderma* SPECIES

### Non‐selectable marker‐dependent genome manipulation

**FIGURE 3:**
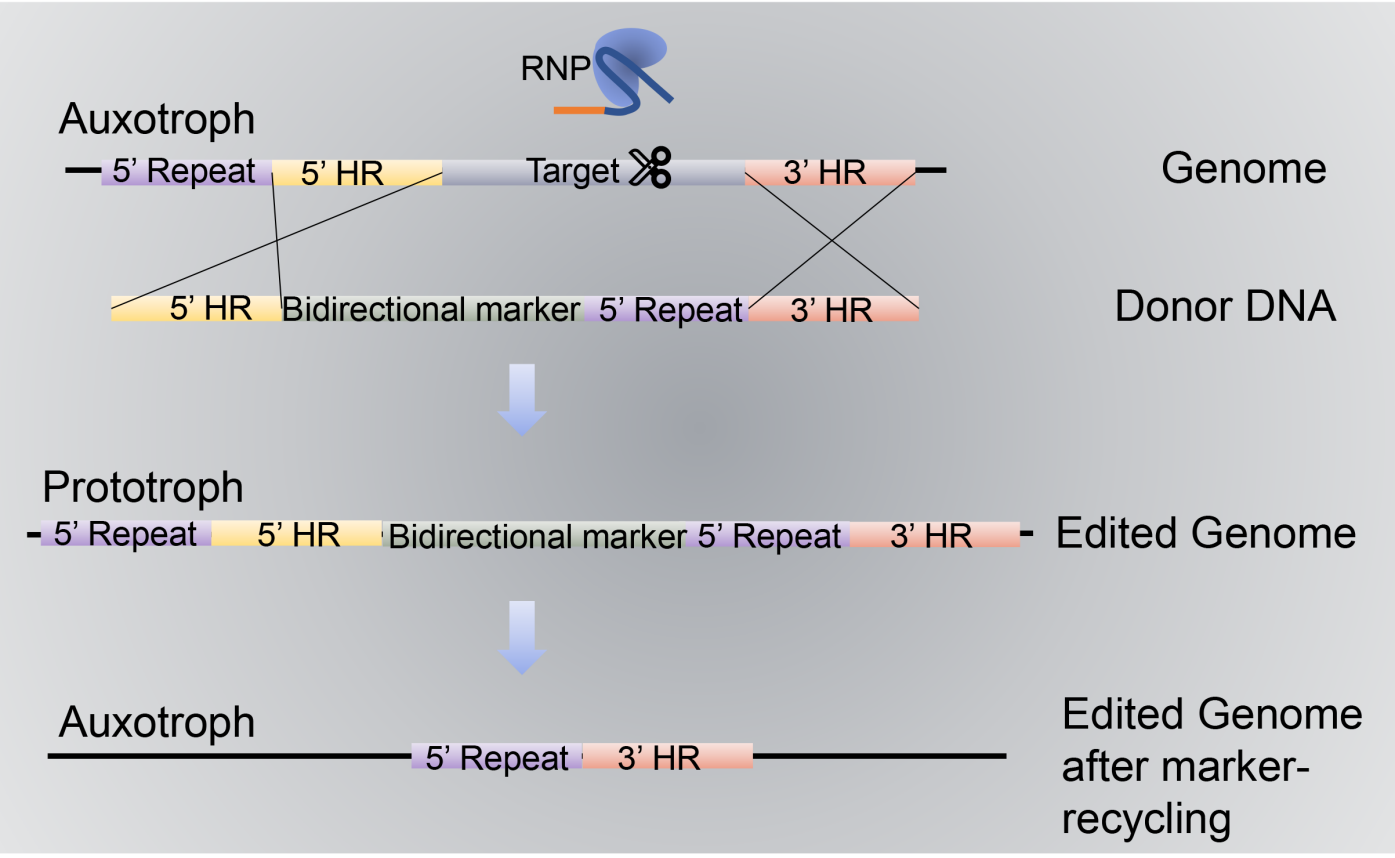
Schematic diagram of marker‐recycling based on uracil auxotrophy. The optimized deletion construct included a bidirectional marker (e.g. *ura5*) (celadon), the 5′ (yellow) and 3′ (pink) flanking region of the target gene (grey), and the 5′ direct repeat (purple), that is, the upstream sequence of the 5′ flanking region. The donor DNA together with the in vitro preassembled RNP was transformed into the auxotrophic strain synchronously to generate edited prototrophic transformants. The bidirectional marker on the transformant genome was recycled via homologous recombination between two direct repeat fragments after incubation on screening plates (e.g. under selection of 5‐FOA). By contrast, transformants with ectopic integration of donor DNA were unable to recycle the bidirectional marker due to a shortage of the essential 5′ direct repeat for homologous recombination.

To create auxotrophic strains for future experiments, the first genes selected by research groups, mostly focusing on *Trichoderma* species, including *T. reesei* (Liu et al., 2015), *T. harzianum* (Vieira et al., 2021), and *T. atroviride* (Primerano, 2021), were the bidirectionally selectable *ura3* and *ura5*, which encode orotidine 5′‐phosphate decarboxylase (URA3) and orotate phosphoribosyl transferase (URA5) (Berges & Barreau, 1991; Table 1). Deletion or mutation of these genes disrupts the pyrimidine biosynthesis pathway, yielding uridine auxotrophic strains (Figure 3). In prototrophic strains, URA5 metabolizes 5‐fuoroorotic acid (5‐FOA), generating 5′ fluorouridine monophosphate, a “suicide” substrate that severely limits cell growth; URA3, which catalyses the second step in the pyrimidine biosynthesis pathway, can also catalyse 5‐FOA to yield the toxic 5′ fluorouridine monophosphate (Berges & Barreau, 1991). Using an RNP‐based CRISPR‐Cas system, auxotrophic strains can be generated without the introduction of exogenous DNA, and it is also possible to edit other loci in the genome by re‐complementing native *ura3*/*ura5* in *T. reesei* (Rantasalo et al., 2019; Zou, Bao et al., 2021; Zou, Li et al., 2021; Zou, Xiao et al., 2021). In addition, the RNP system enabled direct editing of *Trichoderma* strains in the absence of selective pressure; however, the proportion of correctly edited strains remained low (7.37%; Zou, Bao et al., 2021; Zou, Li et al., 2021; Zou, Xiao et al., 2021; Table 1). Importantly, given that these techniques do not involve the transfer of genetic material among organisms, they are not constrained by restrictive GMO‐related regulations, which will be a significant factor in gaining public acceptance of this new biotechnology. However, this is impossible with traditional genetic manipulation techniques.

### RATIONAL DESIGN OF *Trichoderma* FOR INDUSTRIAL PROPERTY OPTIMIZATION

The “working principle” of CRISPR‐Cas will facilitate the genomic engineering of a range of industrially important fungal strains, which will be particularly beneficial, given the paucity of appropriate selective markers and low rates of homologous recombination (Stovicek et al., 2015). In this regard, *T. reesei* is considered a workhorse for industrial cellulase production (Schmoll, 2008), for which CRISPR‐Cas‐based multiplexing genome engineering has been adopted to design a hyper‐producer via direct modification based on the deletion of repressors, overexpression of activators, and introduction of heterologous enzymes with excellent activity (Fonseca et al., 2020; Liu et al., 2015). For example, in the industrially exploited strain *T. reesei* Rut‐C30 (Peterson & Nevalainen, 2012), CRISPR‐Cas was used to engineer the following six genetic modifications: overexpression of the activator XYR1, heterologous β‐glucosidase CEL3A, heterologous invertase SUC1, and deletion of the native repressor ACE1 and secreted proteases PEP1 and SLP1 (Fonseca et al., 2020). These modifications were found to significantly enhance the rate of protein secretion in *T. reesei* Rut‐C30, augmenting inadequate β‐glucosidase production and enhancing sucrose utilization, and alleviates the repressive effects of carbon metabolism. Notably, the modified strain showed enhanced (hemi‐)cellulase activity, with 72‐ and 42‐fold increases in β‐glucosidase and xylanase production, respectively (Fonseca et al., 2020). Given the notable synthesis and secretion characteristics of *T. reesei*, suitably modified strains of this fungus are believed to have considerable promise for the production of large amounts of high value‐added protein. Multiplexed CRISPR‐Cas in combination with a synthetic expression system (SES) enabled the accelerated construction of *T. reesei* strains and increased the production of fully functional calB to 4 g/L without native background enzymes (Rantasalo et al., 2019). In another study, 11 native genes (10 secreted lignocellulose‐degrading enzymes and one protease activator) were selectively deleted using HDR‐stimulated iterative marker recycling (Figure 3), yielding an optimized *T. reesei* chassis that substantially enhanced the production of heterologous genes derived from different organisms (Chai et al., 2022). CRISPR‐Cas makes rational design of strains of *Trichoderma* more feasible and convenient than traditional genetic techniques. However, no progress has been made in the design of strains for agricultural applications.

### Validation of gene (cluster) function

Over the past two decades, new developments in DNA sequencing have enabled the identification of numerous *Trichoderma* genomic sequences. However, the function of the proteins encoded by these sequences or biosynthetic gene clusters of bioactive substances remains to be established (Rush et al., 2021). Even in the most extensively studied *T. reesei*, poor inherent homologous recombination efficiency currently represents a bottleneck constraining advances in functional genomics (Derntl et al., 2016). Fortunately, the CRISPR‐Cas9 system contributes to rapid gene function verification based on bioinformatic annotation. For example, regulation of the putative *Tr*Vib1 protein, an ortholog of *Neurospora crossa Nc*Vib1, was rapidly verified in *T. reesei* using the CRISPR‐Cas system (Liu et al., 2015). Similarly, the combined application of bioinformatics analysis and the CRISPR‐Cas system has contributed to the characterization of a novel specific transcription factor for GH11 xylanase genes (Liu, Chen et al., 2017; Liu, Wang et al., 2017). CRISPR‐Cas was also employed to investigate the causative mutations involved in the reduced viscosity and enhanced volumetric productivity of *T. reesei* mutants with improved industrial fermentation characteristics (Bodie et al., 2021).

Recently, researchers have begun to focus on *Trichoderma* species isolated from specific habitats, which are abundant but rarely investigated fungal sources to produce a wide range of natural products with diverse bioactivities (Liu, Song et al., 2022; Liu, Xu et al., 2022). Moreover, relatively little is known regarding the nature of biosynthetic gene clusters. Thus, the CRISPR‐Cas system would appear to be an ideal platform for mining novel natural products associated with cryptic and uncharacterized biosynthetic gene clusters from these newly isolated *Trichoderma* species (Wang et al., 2022).

### CRISPR‐based regulation in *Trichoderma*

The development of the CRISPR‐Cas system will provide a novel approach for elucidating the mechanisms underlying gene regulation, with biotechnological applications in multiple fields. In addition to investigating the aforementioned functions of transcriptional regulators, CRISPR‐Cas9 offers the prospect of precise gene regulation among the newly developed tools, CRISPR activation (CRISPRa) and CRISPR interference (CRISPRi). In this context, dCas9 (inactive nuclease‐dead mutant) with a gRNA targeting a promoter region has been demonstrated to down‐regulate the expression level of downstream genes, whereas in *Candida albicans*, fusion of dCas to an Mxi1 repressor domain has been found to enhance transcriptional repression (Wensing et al., 2019). In *Pichia pastoris*, CRISPRa/CRISPRi technology has facilitated the more precise regulation of gene expression. Furthermore, when used in conjunction with synthetic promoters in *P. pastoris*, CRISPRa‐based gene expression represents a novel “plug‐and‐play” platform that can be applied to produce customized hosts with high‐level expression that responds to defined signals (Liu, Song et al., 2022; Liu, Xu et al., 2022). However, CRISPRa/CRISPRi systems are yet to be developed for editing the *Trichoderma* genome. It is important to enable partial loss of gene function via precise and quantitative activation/repression, rather than complete loss of gene function. Thus, CRISPRa/CRISPRi is a promising technology for future application in *Trichoderma*.

## FUTURE PERSPECTIVES AND CONCLUSIONS

*Trichoderma* spp. are well‐studied model fungal organisms because of their powerful cellulase productivity and biocontrol properties. Their products include industrial enzyme preparations used in pulp, biofuels, food, feed, and other fields, as well as biofertilizers, biopesticides, and bioremediation agents. In addition to these major applications of *Trichoderma* species, the fields of sustainable green and white biotechnology have become increasingly important for the environmentally safe production of humanized proteins, antibiotics, and other bioactive natural products. Although a number of relevant key factors to improve strain properties have been discovered in recent decades, traditional genetic manipulation has become a bottleneck for further biotechnological exploration of *Trichoderma*.

CRISPR‐Cas genome editing technology enables genetic engineering of a variety of organisms and offers numerous hitherto unattainable strategies for genetic manipulation. Gene‐edited crops and mushrooms have been approved for marketing by several countries and organizations. Notably, the legalization of CRISPR‐Cas‐edited products was realized in less than 5 years following the initial establishment of the CRISPR‐Cas9 concept, thereby highlighting the public's willingness to accept the safety of the technology. Given its appropriate iterative strategy, ease of development, and broad applicability, the CRISPR‐Cas system can undoubtedly be applied in a wide range of biotechnological fields. The newly discovered applications of CRISPR‐Cas in fungal genome editing have unique and powerful capabilities and potential biotechnological applications. Coupled with its efficiency and simplicity, this system will also be broadly applicable in modifying the genomes of little studied and newly isolated strains of *Trichoderma*, and considering its far‐reaching scope, CRISPR‐Cas genome engineering will inevitably expand the application of *Trichoderma* in the fields of industry, agriculture, medicine, and food.

## CONFLICT OF INTEREST

The authors declare that the research was conducted in the absence of any commercial or financial relationships that could be construed as a potential conflict of interest.

## References

[mbt214126-bib-0001] Anzalone, A.V. , Randolph, P.B. , Davis, J.R. , Sousa, A.A. , Koblan, L.W. , Levy, J.M. et al. (2019) Search‐and‐replace genome editing without double‐strand breaks or donor DNA. Nature, 576, 149–157. 31634902 10.1038/s41586-019-1711-4 PMC6907074

[mbt214126-bib-0002] Benitez, T. , Rincon, A.M. , Limon, M.C. & Codon, A.C. (2004) Biocontrol mechanisms of *Trichoderma* strains. International Microbiology, 7, 249–260. 15666245

[mbt214126-bib-0003] Berges, T. & Barreau, C. (1991) Isolation of uridine auxotrophs from *Trichoderma reesei* and efficient transformation with the cloned *ura3* and *ura5* genes. Current Genetics, 19, 359–365. 1913875 10.1007/BF00309596

[mbt214126-bib-0004] Bischof, R.H. , Ramoni, J. & Seiboth, B. (2016) Cellulases and beyond: the first 70 years of the enzyme producer *Trichoderma reesei* . Microbial Cell Factories, 15, 106. 27287427 10.1186/s12934-016-0507-6 PMC4902900

[mbt214126-bib-0005] Bodie, E. , Virag, A. , Pratt, R.J. , Leiva, N. , Ward, M. & Dodge, T. (2021) Reduced viscosity mutants of *Trichoderma reesei* with improved industrial fermentation characteristics. Journal of Industrial Microbiology & Biotechnology, 48, kuab014. 33599729 10.1093/jimb/kuab014 PMC9113505

[mbt214126-bib-0006] Burgess, D.J. (2013) Technology: a CRISPR genome‐editing tool. Nature Reviews. Genetics, 14, 80. 10.1038/nrg3409 23322222

[mbt214126-bib-0007] Cai, F. , Kubicek, C.P. & Druzhinina, I.S. (2021) Genetic transformation of *Trichoderma* spp. Methods in Molecular Biology, 2290, 171–185. 34009590 10.1007/978-1-0716-1323-8_12

[mbt214126-bib-0008] Canzler, S. , Stadler, P.F. & Hertel, J. (2016) U6 snRNA intron insertion occurred multiple times during fungi evolution. RNA Biology, 13, 119–127. 26828373 10.1080/15476286.2015.1132139 PMC4829304

[mbt214126-bib-0009] Cardoza, R.E. , Vizcaino, J.A. , Hermosa, M.R. , Monte, E. & Gutierrez, S. (2006) A comparison of the phenotypic and genetic stability of recombinant *Trichoderma* spp. generated by protoplast‐ and *Agrobacterium*‐mediated transformation. Journal of Microbiology, 44, 383–395. 16953173

[mbt214126-bib-0010] Chai, S. , Zhu, Z. , Tian, E. , Xiao, M. , Wang, Y. , Zou, G. et al. (2022) Building a Versatile Protein Production Platform Using Engineered *Trichoderma reesei* . ACS Synthetic Biology, 11(1), 486–496. 10.1021/acssynbio.1c00570 34928572

[mbt214126-bib-0011] Chen, J. , Lai, Y. , Wang, L. , Zhai, S. , Zou, G. , Zhou, Z. et al. (2017) CRISPR/Cas9‐mediated efficient genome editing via blastospore‐based transformation in entomopathogenic fungus *Beauveria bassiana* . Scientific Reports, 8, 45763. 28368054 10.1038/srep45763 PMC5377935

[mbt214126-bib-0012] Cong, L. , Ran, F.A. , Cox, D. , Lin, S. , Barretto, R. , Habib, N. et al. (2013) Multiplex genome engineering using CRISPR/Cas systems. Science, 339, 819–823. 23287718 10.1126/science.1231143 PMC3795411

[mbt214126-bib-0013] Derntl, C. , Rassinger, A. , Srebotnik, E. , Mach, R.L. & Mach‐Aigner, A.R. (2016) Identification of the main regulator responsible for synthesis of the typical yellow pigment produced by *Trichoderma reesei* . Applied and Environmental Microbiology, 82, 6247–6257. 27520818 10.1128/AEM.01408-16 PMC5068150

[mbt214126-bib-0014] Deveau, H. , Garneau, J.E. & Moineau, S. (2010) CRISPR/Cas system and its role in phage‐bacteria interactions. Annual Review of Microbiology, 64, 475–493. 10.1146/annurev.micro.112408.134123 20528693

[mbt214126-bib-0015] DiCarlo, J.E. , Norville, J.E. , Mali, P. , Rios, X. , Aach, J. & Church, G.M. (2013) Genome engineering in *Saccharomyces cerevisiae* using CRISPR‐Cas systems. Nucleic Acids Research, 41, 4336–4343. 23460208 10.1093/nar/gkt135 PMC3627607

[mbt214126-bib-0016] Dong, M. , Wang, S. , Xu, F. , Xiao, G. , Bai, J. , Wang, J. et al. (2022) Integrative transcriptome and proteome analyses of *Trichoderma longibrachiatum* LC and its cellulase hyper‐producing mutants generated by heavy ion mutagenesis reveal the key genes involved in cellulolytic enzymes regulation. Biotechnology for Biofuels and Bioproducts, 15, 63. 35658919 10.1186/s13068-022-02161-7 PMC9166314

[mbt214126-bib-0017] Druzhinina, I.S. , Chenthamara, K. , Zhang, J. , Atanasova, L. , Yang, D. , Miao, Y. et al. (2018) Massive lateral transfer of genes encoding plant cell wall‐degrading enzymes to the mycoparasitic fungus *Trichoderma* from its plant‐associated hosts. PLoS Genetics, 14, e1007322. 29630596 10.1371/journal.pgen.1007322 PMC5908196

[mbt214126-bib-0018] Enkler, L. , Richer, D. , Marchand, A.L. , Ferrandon, D. & Jossinet, F. (2016) Genome engineering in the yeast pathogen *Candida glabrata* using the CRISPR‐Cas9 system. Scientific Reports, 6, 35766. 27767081 10.1038/srep35766 PMC5073330

[mbt214126-bib-0019] Fonseca, L.M. , Parreiras, L.S. & Murakami, M.T. (2020) Rational engineering of the *Trichoderma reesei* RUT‐C30 strain into an industrially relevant platform for cellulase production. Biotechnology for Biofuels, 13, 93. 32461765 10.1186/s13068-020-01732-w PMC7243233

[mbt214126-bib-0020] Foster, A.J. , Martin‐Urdiroz, M. , Yan, X. , Wright, H.S. , Soanes, D.M. & Talbot, N.J. (2018) CRISPR‐Cas9 ribonucleoprotein‐mediated co‐editing and counterselection in the rice blast fungus. Scientific Reports, 8, 14355. 30254203 10.1038/s41598-018-32702-w PMC6156577

[mbt214126-bib-0021] Gajera, H.P. , Hirpara, D.G. , Katakpara, Z.A. , Patel, S.V. & Golakiya, B.A. (2016) Molecular evolution and phylogenetic analysis of biocontrol genes acquired from SCoT polymorphism of mycoparasitic *Trichoderma koningii* inhibiting phytopathogen *Rhizoctonia solani* Kuhn. Infection, Genetics and Evolution, 45, 383–392. 10.1016/j.meegid.2016.09.026 27720889

[mbt214126-bib-0022] Goldman, G.H. , Van Montagu, M. & Herrera‐Estrella, A. (1990) Transformation of *Trichoderma harzianum* by high‐voltage electric pulse. Current Genetics, 17, 169–174.

[mbt214126-bib-0023] de Groot, M.J. , Bundock, P. , Hooykaas, P.J. & Beijersbergen, A.G. (1998) *Agrobacterium tumefaciens*‐mediated transformation of filamentous fungi. Nature Biotechnology, 16, 839–842. 10.1038/nbt0998-839 9743116

[mbt214126-bib-0024] Hao, Z. & Su, X. (2019) Fast gene disruption in *Trichoderma reesei* using *in vitro* assembled Cas9/gRNA complex. BMC Biotechnology, 19, 2. 30626373 10.1186/s12896-018-0498-y PMC6325762

[mbt214126-bib-0025] Hazell, B.W. , Te'o, V.S. , Bradner, J.R. , Bergquist, P.L. & Nevalainen, K.M. (2000) Rapid transformation of high cellulase‐producing mutant strains of *Trichoderma reesei* by microprojectile bombardment. Letters in Applied Microbiology, 30, 282–286. 10792647 10.1046/j.1472-765x.2000.00715.x

[mbt214126-bib-0026] Herrera‐Estrella, A. , Goldman, G.H. & Van Montagu, M. (1990) High‐efficiency transformation system for the biocontrol agents, *Trichoderma* spp. Molecular Microbiology, 4, 839–843. 2388561 10.1111/j.1365-2958.1990.tb00654.x

[mbt214126-bib-0027] Horwitz, A.A. , Walter, J.M. , Schubert, M.G. , Kung, S.H. , Hawkins, K. , Platt, D.M. et al. (2015) Efficient multiplexed integration of synergistic alleles and metabolic pathways in yeasts via CRISPR‐Cas. Cell Systems, 1, 88–96. 27135688 10.1016/j.cels.2015.02.001

[mbt214126-bib-0028] Jiang, D. , Zhu, W. , Wang, Y. , Sun, C. , Zhang, K.Q. & Yang, J. (2013) Molecular tools for functional genomics in filamentous fungi: recent advances and new strategies. Biotechnology Advances, 31, 1562–1574. 23988676 10.1016/j.biotechadv.2013.08.005

[mbt214126-bib-0029] Jiang, Y. , Qian, F. , Yang, J. , Liu, Y. , Dong, F. , Xu, C. et al. (2017) CRISPR‐Cpf1 assisted genome editing of *Corynebacterium glutamicum* . Nature Communications, 8, 15179. 10.1038/ncomms15179 PMC5418603 28469274

[mbt214126-bib-0030] Kanchiswamy, C.N. (2016) DNA‐free genome editing methods for targeted crop improvement. Plant Cell Reports, 35, 1469–1474. 27100964 10.1007/s00299-016-1982-2

[mbt214126-bib-0031] Kang, M. , Zuo, Z. , Yin, Z. & Gu, J. (2022) Molecular mechanism of D1135E‐induced discriminated CRISPR‐Cas9 PAM recognition. Journal of Chemical Information and Modeling, 62, 3057–3066. 35666156 10.1021/acs.jcim.1c01562

[mbt214126-bib-0032] Kaya, H. , Mikami, M. , Endo, A. , Endo, M. & Toki, S. (2016) Highly specific targeted mutagenesis in plants using *Staphylococcus aureus* Cas9. Scientific Reports, 6, 26871. 27226350 10.1038/srep26871 PMC4881040

[mbt214126-bib-0033] Kim, S. , and Miasnikov, A. (2013) Method for introducing nucleic acids into fungal cells . US Patent, US8450098B2.

[mbt214126-bib-0034] Kim, S. , Kim, D. , Cho, S.W. , Kim, J. & Kim, J.S. (2014) Highly efficient RNA‐guided genome editing in human cells via delivery of purified Cas9 ribonucleoproteins. Genome Research, 24, 1012–1019. 24696461 10.1101/gr.171322.113 PMC4032847

[mbt214126-bib-0035] Kubicek, C.P. , Herrera‐Estrella, A. , Seidl‐Seiboth, V. , Martinez, D.A. , Druzhinina, I.S. , Thon, M. et al. (2011) Comparative genome sequence analysis underscores mycoparasitism as the ancestral life style of Trichoderma. Genome Biology, 12, R40. 21501500 10.1186/gb-2011-12-4-r40 PMC3218866

[mbt214126-bib-0036] Kubicek, C.P. , Steindorff, A.S. , Chenthamara, K. , Manganiello, G. , Henrissat, B. , Zhang, J. et al. (2019) Evolution and comparative genomics of the most common *Trichoderma* species. BMC Genomics, 20, 485. 31189469 10.1186/s12864-019-5680-7 PMC6560777

[mbt214126-bib-0037] Kumar, S. , Shukla, V. , Dubey, M.K. & Upadhyay, R.S. (2021) Activation of defense response in common bean against stem rot disease triggered by *Trichoderma erinaceum* and *Trichoderma viride* . Journal of Basic Microbiology, 61, 910–922. 34398489 10.1002/jobm.202000749

[mbt214126-bib-0038] Lee, Y. , Kim, M. , Han, J. , Yeom, K.H. , Lee, S. , Baek, S.H. et al. (2004) MicroRNA genes are transcribed by RNA polymerase II. The EMBO Journal, 23, 4051–4060. 15372072 10.1038/sj.emboj.7600385 PMC524334

[mbt214126-bib-0039] Li, W. , Teng, F. , Li, T. & Zhou, Q. (2013) Simultaneous generation and germline transmission of multiple gene mutations in rat using CRISPR‐Cas systems. Nature Biotechnology, 31, 684–686. 10.1038/nbt.2652 23929337

[mbt214126-bib-0040] Li, D. , Tang, Y. , Lin, J. & Cai, W. (2017) Methods for genetic transformation of filamentous fungi. Microbial Cell Factories, 16, 168. 28974205 10.1186/s12934-017-0785-7 PMC5627406

[mbt214126-bib-0041] Li, W.C. , Lin, T.C. , Chen, C.L. , Liu, H.C. , Lin, H.N. , Chao, J.L. et al. (2021) Complete genome sequences and genome‐wide characterization of *Trichoderma* biocontrol agents provide new insights into their evolution and variation in genome organization, sexual development, and fungal‐plant interactions. Microbiology Spectrum, 9, e0066321. 34908505 10.1128/Spectrum.00663-21 PMC8672877

[mbt214126-bib-0042] Li, H. , Liu, X. , Li, X. , Hu, Z. & Wang, L. (2021) Novel harziane diterpenes from deep‐sea sediment fungus *Trichoderma* sp SCSIOW21 and their potential anti‐inflammatory effects. Marine Drugs, 19, 689. 34940688 10.3390/md19120689 PMC8705903

[mbt214126-bib-0043] Liu, L. & Fan, X.D. (2014) CRISPR‐Cas system: a powerful tool for genome engineering. Plant Molecular Biology, 85, 209–218. 24639266 10.1007/s11103-014-0188-7

[mbt214126-bib-0044] Liu, R. , Chen, L. , Jiang, Y. , Zhou, Z. & Zou, G. (2015) Efficient genome editing in filamentous fungus *Trichoderma reesei* using the CRISPR/Cas9 system. Cell Discovery, 1, 15007. 27462408 10.1038/celldisc.2015.7 PMC4860831

[mbt214126-bib-0045] Liu, R. , Chen, L. , Jiang, Y. , Zou, G. & Zhou, Z. (2017) A novel transcription factor specifically regulates GH11 xylanase genes in *Trichoderma reesei* . Biotechnology for Biofuels, 10, 194. 28785310 10.1186/s13068-017-0878-x PMC5541735

[mbt214126-bib-0046] Liu, P. , Wang, W. & Wei, D. (2017) Use of transcription activator‐like effector for efficient gene modification and transcription in the filamentous fungus *Trichoderma reesei* . Journal of Industrial Microbiology & Biotechnology, 44, 1367–1373. 28674932 10.1007/s10295-017-1963-7

[mbt214126-bib-0047] Liu, Q. , Zhang, Y. , Li, F. , Li, J. , Sun, W. & Tian, C. (2019) Upgrading of efficient and scalable CRISPR‐Cas‐mediated technology for genetic engineering in thermophilic fungus *Myceliophthora thermophila* . Biotechnology for Biofuels, 12, 293. 31890021 10.1186/s13068-019-1637-y PMC6927189

[mbt214126-bib-0048] Liu, Q. , Song, L. , Peng, Q. , Zhu, Q. , Shi, X. , Xu, M. et al. (2022) A programmable high‐expression yeast platform responsive to user‐defined signals. Science Advances, 8, eabl5166. 35148182 10.1126/sciadv.abl5166 PMC8836803

[mbt214126-bib-0049] Liu, S.Z. , Xu, G.X. , He, F.M. , Zhang, W.B. , Wu, Z. , Li, M.Y. et al. (2022) New sorbicillinoids with tea pathogenic fungus inhibitory effect from marine‐derived fungus *Hypocrea jecorina* H8. Marine Drugs, 20(3), 213. 10.3390/md20030213 PMC8955853 35323512

[mbt214126-bib-0050] Lorito, M. , Hayes, C.K. , Di Pietro, A. & Harman, G.E. (1993) Biolistic transformation of *Trichoderma harzianum* and *Gliocladium virens* using plasmid and genomic DNA. Current Genetics, 24, 349–356. 8252645 10.1007/BF00336788

[mbt214126-bib-0051] Martinez, D. , Berka, R.M. , Henrissat, B. , Saloheimo, M. , Arvas, M. , Baker, S.E. et al. (2008) Genome sequencing and analysis of the biomass‐degrading fungus *Trichoderma reesei* (syn. *Hypocrea jecorina*). Nature Biotechnology, 26, 553–560. 10.1038/nbt1403 18454138

[mbt214126-bib-0052] Michielse, C.B. , Ram, A.F. , Hooykaas, P.J. & van den Hondel, C.A. (2004) *Agrobacterium*‐mediated transformation of *Aspergillus awamori* in the absence of full‐length VirD2, VirC2, or VirE2 leads to insertion of aberrant T‐DNA structures. Journal of Bacteriology, 186, 2038–2045. 15028687 10.1128/JB.186.7.2038-2045.2004 PMC374399

[mbt214126-bib-0053] Mukherjee, P.K. , Horwitz, B.A. , Herrera‐Estrella, A. , Schmoll, M. & Kenerley, C.M. (2013) *Trichoderma* research in the genome era. Annual Review of Phytopathology, 51, 105–129. 10.1146/annurev-phyto-082712-102353 23915132

[mbt214126-bib-0054] Papzan, Z. , Kowsari, M. , Javan‐Nikkhah, M. , Gohari, A.M. & Limon, M.C. (2021) Strain improvement of *Trichoderma* spp. through two‐step protoplast fusion for cellulase production enhancement. Canadian Journal of Microbiology, 67, 406–414. 33226848 10.1139/cjm-2020-0438

[mbt214126-bib-0055] Paul, B. & Montoya, G. (2020) CRISPR‐Cas12a: Functional overview and applications. Biomed J, 43, 8–17. 32200959 10.1016/j.bj.2019.10.005 PMC7090318

[mbt214126-bib-0056] Penttilaa, M. , Nevalainen, H. , Ratto, M. , Salminen, E. & Knowles, J. (1987) A versatile transformation system for the cellulolytic filamentous fungus *Trichoderma reesei* . Gene, 61, 155–164. 3127274 10.1016/0378-1119(87)90110-7

[mbt214126-bib-0057] Peterson, R. & Nevalainen, H. (2012) *Trichoderma reesei* RUT‐C30‐‐thirty years of strain improvement. Microbiology, 158, 58–68. 21998163 10.1099/mic.0.054031-0

[mbt214126-bib-0058] Primerano, P. (2021) Genetic manipulations in *Trichoderma reesei* and *Trichoderma atroviride*–Auxotrophic selection markers and the CRISPR/Cas9 system. Master thesis. Vienna University of Technology, Austria.

[mbt214126-bib-0059] Qin, H. , Xiao, H. , Zou, G. , Zhou, Z.H. & Zhong, J.J. (2017) CRISPR‐Cas9 assisted gene disruption in the higher fungus *Ganoderma* species. Process Biochemistry, 56, 57–61.

[mbt214126-bib-0060] Rantasalo, A. , Vitikainen, M. , Paasikallio, T. , Jantti, J. , Landowski, C.P. & Mojzita, D. (2019) Novel genetic tools that enable highly pure protein production in *Trichoderma reesei* . Scientific Reports, 9, 5032. 30902998 10.1038/s41598-019-41573-8 PMC6430808

[mbt214126-bib-0061] Rush, T.A. , Shrestha, H.K. , Gopalakrishnan Meena, M. , Spangler, M.K. , Ellis, J.C. , Labbé, J.L. et al. (2021) Bioprospecting Trichoderma: a systematic roadmap to screen genomes and natural products for biocontrol applications. Frontiers in Fungal Biology, 2, 716511. 10.3389/ffunb.2021.716511 PMC10512312 37744103

[mbt214126-bib-0062] Sanchez‐Torres, P. , Gonzalez, R. , Perez‐Gonzalez, J.A. , Gonzalez‐Candelas, L. & Ramon, D. (1994) Development of a transformation system for *Trichoderma longibrachiatum* and its use for constructing multicopsy transformants for the *egl1* gene. Applied Microbiology and Biotechnology, 41, 440–446. 7765105 10.1007/BF00939033

[mbt214126-bib-0063] Schmoll, M. (2008) The information highways of a biotechnological workhorse – signal transduction in *Hypocrea jecorina* . BMC Genomics, 9, 430. 18803869 10.1186/1471-2164-9-430 PMC2566311

[mbt214126-bib-0064] Schuster, M. & Kahmann, R. (2019) CRISPR‐Cas9 genome editing approaches in filamentous fungi and oomycetes. Fungal Genetics and Biology, 130, 43–53. 31048007 10.1016/j.fgb.2019.04.016

[mbt214126-bib-0065] de Souza Maia Filho, F. , da Silva Fonseca, A.O. , Persici, B.M. , de Souza Silveira, J. , Braga, C.Q. , Potter, L. et al. (2017) *Trichoderma virens* as a biocontrol of *Toxocara canis*: *In vivo* evaluation. Revista iberoamericana de micologia, 34, 32–35. 28109772 10.1016/j.riam.2016.06.004

[mbt214126-bib-0066] Sridharan, A.P. , Sugitha, T. , Karthikeyan, G. , Nakkeeran, S. & Sivakumar, U. (2021) Metabolites of *Trichoderma longibrachiatum* EF5 inhibits soil borne pathogen, *Macrophomina phaseolin*a by triggering amino sugar metabolism. Microbial Pathogenesis, 150, 104714. 33383148 10.1016/j.micpath.2020.104714

[mbt214126-bib-0067] Steindorff, A.S. , Ramada, M.H. , Coelho, A.S. , Miller, R.N. , Pappas, G.J., Jr. , Ulhoa, C.J. et al. (2014) Identification of mycoparasitism‐related genes against the phytopathogen *Sclerotinia sclerotiorum* through transcriptome and expression profile analysis in *Trichoderma harzianum* . BMC Genomics, 15, 204. 24635846 10.1186/1471-2164-15-204 PMC4004048

[mbt214126-bib-0068] Stovicek, V. , Borodina, I. & Forster, J. (2015) CRISPR‐Cas system enables fast and simple genome editing of industrial *Saccharomyces cerevisiae* strains. Metabolic Engineering Communications, 2, 13–22. 34150504 10.1016/j.meteno.2015.03.001 PMC8193243

[mbt214126-bib-0069] TariqJaveed, M. , Farooq, T. , Al‐Hazmi, A.S. , Hussain, M.D. & Rehman, A.U. (2021) Role of *Trichoderma* as a biocontrol agent (BCA) of phytoparasitic nematodes and plant growth inducer. Journal of Invertebrate Pathology, 183, 107626. 34081963 10.1016/j.jip.2021.107626

[mbt214126-bib-0070] Te'o, V.S. , Bergquist, P.L. & Nevalainen, K.M. (2002) Biolistic transformation of *Trichoderma reesei* using the Bio‐Rad seven barrels Hepta Adaptor system. Journal of Microbiological Methods, 51, 393–399. 12223300 10.1016/s0167-7012(02)00126-4

[mbt214126-bib-0071] Tomico‐Cuenca, I. , Mach, R.L. , Mach‐Aigner, A.R. & Derntl, C. (2021) An overview on current molecular tools for heterologous gene expression in *Trichoderma* . Fungal Biology and Biotechnology, 8, 11. 34702369 10.1186/s40694-021-00119-2 PMC8549263

[mbt214126-bib-0072] Vidgren, V. , Halinen, S. , Tamminen, A. , Olenius, S. & Wiebe, M.G. (2020) Engineering marine fungi for conversion of D‐galacturonic acid to mucic acid. Microbial Cell Factories, 19, 156. 32736636 10.1186/s12934-020-01411-3 PMC7393721

[mbt214126-bib-0073] Vieira, A.A. , Vianna, G.R. , Carrijo, J. , Aragao, F.J.L. & Vieira, P.M. (2021) Generation of *Trichoderma harzianum* with *pyr4* auxotrophic marker by using the CRISPR/Cas9 system. Scientific Reports, 11, 1085. 33441796 10.1038/s41598-020-80186-4 PMC7806921

[mbt214126-bib-0074] Wang, Q. , Zhao, Q. , Liu, Q. , He, X. , Zhong, Y. , Qin, Y. et al. (2021) CRISPR/Cas9‐mediated genome editing in *Penicillium oxalicum* and *Trichoderma reesei* using 5S rRNA promoter‐driven guide RNAs. Biotechnology Letters, 43, 495–502. 33048255 10.1007/s10529-020-03024-7

[mbt214126-bib-0075] Wang, Y. , Li, X.M. , Yang, S.Q. , Zhang, F.Z. , Wang, B.G. , Li, H.L. et al. (2022) Sesquiterpene and Sorbicillinoid glycosides from the endophytic fungus *Trichoderma longibrachiatum* EN‐586 derived from the marine Red Alga Laurencia obtusa. Marine Drugs, 20, 177. 35323476 10.3390/md20030177 PMC8949086

[mbt214126-bib-0076] Wensing, L. , Sharma, J. , Uthayakumar, D. , Proteau, Y. , Chavez, A. & Shapiro, R.S. (2019) A CRISPR interference platform for efficient genetic repression in *Candida albicans* . mSphere, 4(1), e00002‐19. 10.1128/mSphere.00002-19 PMC6374589 30760609

[mbt214126-bib-0077] Wu, C. , Chen, Y. , Huang, X. , Sun, S. , Luo, J. , Lu, Z. et al. (2020) An efficient shortened genetic transformation strategy for filamentous fungus *Trichoderma reesei* . The Journal of General and Applied Microbiology, 65, 301–307. 31231078 10.2323/jgam.2019.02.001

[mbt214126-bib-0078] Wu, C. , Chen, Y. , Qiu, Y. , Niu, X. , Zhu, N. , Chen, J. et al. (2020) A simple approach to mediate genome editing in the filamentous fungus *Trichoderma reesei* by CRISPR/Cas9‐coupled *in vivo* gRNA transcription. Biotechnology Letters, 42, 1203–1210. 32300998 10.1007/s10529-020-02887-0

[mbt214126-bib-0079] Xian, H.Q. , Liu, L. , Li, Y.H. , Yang, Y.N. & Yang, S. (2020) Molecular tagging of biocontrol fungus *Trichoderma asperellum* and its colonization in soil. Journal of Applied Microbiology, 128, 255–264. 31541488 10.1111/jam.14457

[mbt214126-bib-0080] Xie, L. , Zang, X. , Cheng, W. , Zhang, Z. , Zhou, J. , Chen, M. et al. (2021) Harzianic acid from *Trichoderma afroharzianum* is a natural product inhibitor of acetohydroxyacid synthase. Journal of the American Chemical Society., 143, 9575–9584. 10.1021/jacs.1c03988 PMC8674378 34132537

[mbt214126-bib-0081] Yu, L. , Xiao, M. , Zhu, Z. , Wang, Y. , Zhou, Z. , Wang, P. et al. (2022) Efficient genome editing in *Claviceps purpurea* using a CRISPR/Cas9 ribonucleoprotein method. Synthetic and Systems Biotechnology, 7, 664–670. 35224234 10.1016/j.synbio.2022.02.002 PMC8857428

[mbt214126-bib-0082] Zain Ul Arifeen, M. , Ma, Y.N. , Xue, Y.R. & Liu, C.H. (2019) Deep‐sea fungi could be the new arsenal for bioactive molecules. Marine Drugs, 18, 9. 10.3390/md18010009 PMC7024341 31861953

[mbt214126-bib-0083] Zavala‐Gonzalez, E.A. , Lopez‐Moya, F. , Aranda‐Martinez, A. , Cruz‐Valerio, M. , Lopez‐Llorca, L.V. & Ramirez‐Lepe, M. (2016) Tolerance to chitosan by *Trichoderma* species is associated with low membrane fluidity. Journal of Basic Microbiology, 56, 792–800. 27213758 10.1002/jobm.201500758

[mbt214126-bib-0084] Zeilinger, S. (2004) Gene disruption in *Trichoderma atroviride* via *Agrobacterium*‐mediated transformation. Current Genetics, 45, 54–60. 14586554 10.1007/s00294-003-0454-8

[mbt214126-bib-0085] Zeng, R. , Yin, X.Y. , Ruan, T. , Hu, Q. , Hou, Y.L. , Zuo, Z.Y. et al. (2016) A novel cellulase produced by a newly isolated *Trichoderma virens* . Bioengineering (Basel, Switzerland), 3, 13. 10.3390/bioengineering3020013 PMC5597137 28952575

[mbt214126-bib-0086] Zhang, F. , Ge, H. , Zhang, F. , Guo, N. , Wang, Y. , Chen, L. et al. (2016) Biocontrol potential of *Trichoderma harzianum* isolate T‐aloe against *Sclerotinia sclerotiorum* in soybean. Plant Physiology and Biochemistry, 100, 64–74. 26774866 10.1016/j.plaphy.2015.12.017

[mbt214126-bib-0087] Zhang, J.L. , Tang, W.L. , Huang, Q.R. , Li, Y.Z. , Wei, M.L. , Jiang, L.L. et al. (2021) *Trichoderma*: a treasure house of structurally diverse secondary metabolites with medicinal importance. Frontiers in Microbiology, 12, 723828. 34367122 10.3389/fmicb.2021.723828 PMC8342961

[mbt214126-bib-0088] Zhang, C. , Li, N. , Rao, L. , Li, J. , Liu, Q. & Tian, C. (2022) Development of an efficient C‐to‐T base‐editing system and its application to cellulase transcription factor precise engineering in thermophilic fungus *Myceliophthora thermophila* . Microbiology Spectrum, 10, e0232121. 35608343 10.1128/spectrum.02321-21 PMC9241923

[mbt214126-bib-0089] Zheng, Y.M. , Lin, F.L. , Gao, H. , Zou, G. , Zhang, J.W. , Wang, G.Q. et al. (2017) Development of a versatile and conventional technique for gene disruption in filamentous fungi based on CRISPR‐Cas9 technology. Scientific Reports, 7, 9250. 28835711 10.1038/s41598-017-10052-3 PMC5569088

[mbt214126-bib-0090] Zhong, Y.H. , Wang, X.L. , Wang, T.H. & Jiang, Q. (2007) *Agrobacterium*‐mediated transformation (AMT) of *Trichoderma reesei* as an efficient tool for random insertional mutagenesis. Applied Microbiology and Biotechnology, 73, 1348–1354. 17021875 10.1007/s00253-006-0603-3

[mbt214126-bib-0091] Zou, G. & Zhou, Z. (2021) CRISPR/Cas9‐mediated genome editing of *Trichoderma reesei* . Methods in Molecular Biology (Clifton, N.J.), 2234, 87–98. 10.1007/978-1-0716-1048-0_8 33165782

[mbt214126-bib-0092] Zou, G. , Bao, D. , Wang, Y. , Zhou, S. , Xiao, M. , Yang, Z. et al. (2021) Alleviating product inhibition of *Trichoderma reesei* cellulase complex with a product‐activated mushroom endoglucanase. Bioresource Technology, 319, 124119. 32957048 10.1016/j.biortech.2020.124119

[mbt214126-bib-0093] Zou, G. , Li, B. , Wang, Y. , Yin, X. , Gong, M. , Shang, J. et al. (2021) Efficient conversion of spent mushroom substrate into a high value‐added anticancer drug pentostatin with engineered *Cordyceps militaris* . Green Chemistry, 23, 10030–10038.

[mbt214126-bib-0094] Zou, G. , Xiao, M. , Chai, S. , Zhu, Z. , Wang, Y. & Zhou, Z. (2021) Efficient genome editing in filamentous fungi via an improved CRISPR‐Cas9 ribonucleoprotein method facilitated by chemical reagents. Microbial Biotechnology, 14, 2343–2355. 32841542 10.1111/1751-7915.13652 PMC8601184

